# Actin‐templated Structures: Nature's Way to Hierarchical Surface Patterns (Gecko's Setae as Case Study)

**DOI:** 10.1002/advs.202303816

**Published:** 2023-12-25

**Authors:** Jennifer Y. Kasper, Matthias W. Laschke, Marcus Koch, Lorenzo Alibardi, Thomas Magin, Carien M. Niessen, Aránzazu del Campo

**Affiliations:** ^1^ INM‐Leibniz Institute for New Materials Campus D2 2 66123 Saarbruecken Germany; ^2^ Institute for Clinical and Experimental Surgery Saarland University 66421 Homburg Germany; ^3^ Comparative Anatomy Department of Biology University of Bologna & Comparative Histolab 40126 Bologna Italy; ^4^ Division of Cell and Developmental Biology Institute of Biology Leipzig University 04103 Leipzig Germany; ^5^ Department Cell Biology of the Skin Cologne Excellence Cluster for Stress Responses in Ageing‐associated diseases (CECAD) Center for Molecular Medicine Cologne (CMMC) University Hospital Cologne University of Cologne 50931 Cologne Germany; ^6^ Chemistry Department Saarland University 66123 Saarbruecken Germany

**Keywords:** actin assembly, apical topographies, cornified tissue, gecko‐inspired adhesives, keratin assembly, setae, surface patterns

## Abstract

The hierarchical design of the toe pad surface in geckos and its reversible adhesiveness have inspired material scientists for many years. Micro‐ and nano‐patterned surfaces with impressive adhesive performance have been developed to mimic gecko's properties. While the adhesive performance achieved in some examples has surpassed living counterparts, the durability of the fabricated surfaces is limited and the capability to self‐renew and restore function—inherent to biological systems—is unimaginable. Here the morphogenesis of gecko setae using skin samples from the Bibron´s gecko (*Chondrodactylus bibronii*) is studied. Gecko setae develop as specialized apical differentiation structures at a distinct cell–cell layer interface within the skin epidermis. A primary role for F‐actin and microtubules as templating structural elements is necessary for the development of setae's hierarchical morphology, and a stabilization role of keratins and corneus beta proteins is identified. Setae grow from single cells in a bottom layer protruding into four neighboring cells in the upper layer. The resulting multicellular junction can play a role during shedding by facilitating fracture of the cell–cell interface and release of the high aspect ratio setae. The results contribute to the understanding of setae regeneration and may inspire future concepts to bioengineer self‐renewable patterned adhesive surfaces.

## Graphical abstract

Gecko‐inspired reversible adhesive surfaces achieve impressive adhesive performance, but have neither a self‐repair, nor a self‐growth mechanism or zero‐waste disposal like the natural tissue. This article studies how the adhesive features on the surface of gecko toe pads (setae) form and mature during a renewal cycle. These studies contribute to the understanding of the morphogenesis of high‐performance natural materials and can inspire new ways to bioengineer materials with unprecedented properties.

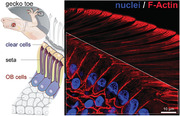

## 1 Introduction

Epithelial cells develop apical protrusions to fulfill diverse functions adapted to the tissue and organismal needs. Microvilli in intestinal epithelium for absorption, stereocilia in cochlear hair cells for mechanosensing, or microridges on mucosal epithelium for mucus retention and transport functions, are representative examples of apical protrusions.^[^ ^1^ ^]^ The advanced properties of these natural surface structures have inspired the design of surface patterns in synthetic materials for decades, and several artificial mimics have been reported.^[^ ^2^ ^]^

To maintain function during the lifetime of the organism, epithelial layers undergo cycles of self‐renewal including shedding, growth, and differentiation. The renewal of the functional apical protrusions is also part of this cycle.^[^ ^3^ ^]^ In contrast to the self‐renewal of epithelial surface patterns, restoration of function in bioinspired surface patterns typically requires manufacturing a new surface layer (or a new whole device) in a multistep, top‐down nanomanufacturing chain and generates waste. The vision to take nature's models a step further and apply bioengineering concepts to pattern material surfaces with self‐renewable layers is appealing from a functional and a sustainability perspective. Although currently unattainable, a profound understanding of self‐renewal in natural surface patterns could enable us in the future to recapitulate a self‐patterning ability in biohybrid coatings or reconstituted cell systems.

The hairy structure on the lizard's toe pad skin represents a unique example of epithelial differentiation and replacement^[^ ^4^ ^]^ that provides the toe pad skin with lifelong unique reversible adhesive properties^[^ ^5^ ^]^ and self‐cleaning capability.^[^ ^6^ ^]^ The sophisticated structure of the most apical structures, called setae,^[^ ^7^ ^]^ is the basis for these properties. Setae are high aspect ratio microfibrils with branched nanosized terminals and triangular‐shaped tips known as spatulae. Live long renewal is driven by the cyclic formation of a new epidermis (inner epidermal generation) underneath the old epidermis (outer epidermal generation), where at the interface between these two epidermal generations, the new surface pattern (setae arrays) is formed due to a concerted interplay between the bottom layer of the outer epidermal generation (the clear cell layer) and the upper layer of the inner generation (the Oberhäutchen layer). When the worn, outer epidermal layer is shed, the underlying new epidermis already has mature apical structures to ensure life‐long reversible adhesive function.

The formation of epithelial projections in human tissues has been studied from the perspective of membrane structures formed by self‐assembly of cytoskeletal components (filamentous actin and microtubules), which actively deform the cell membrane and stabilize the protruding apical structure assisted by anchor and crosslinker proteins.^[^ ^3^ ^]^ In contrast, setae have mainly been studied as cornified structures with a focus on the type and nature of the involved keratin proteins and the resulting mechanical and adhesive properties.^[^ ^8^ ^]^ Mature setae are known to be composed of corneous beta proteins (CBPs, formerly known as β‐keratins) and α‐keratins, and they are covered by stacked lipid layers.^[^ ^9^ ^]^ Keratins, as intermediate filaments, form filamentous network‐like structures in the cellular cytosol,^[^ ^10^ ^]^ but how do keratins organize into such high aspect ratio and hierarchical protrusions? As apical differentiation structures, a contribution of other cytoskeletal components is expected in setae formation.^[^ ^4a^ ^]^

In this work, we have analyzed the cytoskeleton organization in skin samples from the Bibron´s gecko (*Chondrodactylus bibronii*), which are close to the endpoint of the shedding cycle and display old and new epidermal generations in their skin. We have identified fundamental features of setae morphogenesis as apical protrusions at a distinct cell–cell interface. Our findings reveal how a natural surface pattern self‐renews and could be relevant for bioengineering‐based approaches to functional materials synthesis in the future.

## 2 Results

### 2.1 Morphological and Compositional Features of Gecko's setae

**Figure 1:**
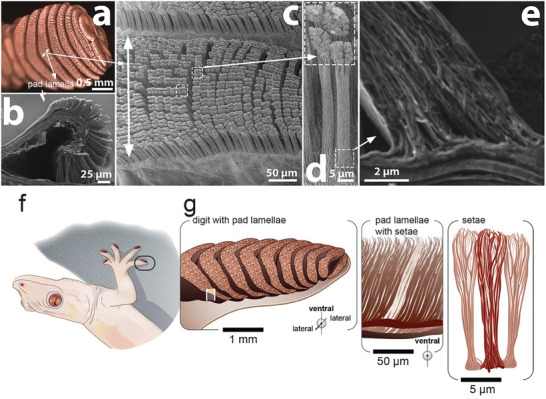
a–e) Images of the epidermal tissue of the toe pad of a young adult Bibron's thick‐toed gecko (*Chondrodactylus bibronii*). a) Ventral side of a gecko toe with toe pad lamellae imaged with a stereomicroscope. b) Scanning electron micrograph (SEM) of a longitudinal dissected toe. c) SEM top view on a pad lamella. The dashed boxes highligh the arrangement of setae in packs of four. d) SEM side view of mature setae. e) SEM image from a longitudinally cryosectioned setal base. The fibers emerge from the base and align along the setae length. f, g) Scheme of a gecko with a marked toe magnified in (g) and depicting the pad lamellae and the setae structures. Illustration by IlluScientia, Dr. Agnieszka Kawska.

The hierarchical surface design of a lizard's toe pad has been reported for different gecko species.^[^ ^7^, ^11^ ^]^ We summarize here the essential features observed in the ventral surface of the toe pad of a young adult Bibron´s thick‐toed gecko used in our study (**Figure** **1**a–g). The toe pad (3.25 mm long, 2.50 mm wide) is covered by 10 lamellae arranged in parallel along the transversal direction (Figure 1a). Hair‐like appendages protrude from the surface (Figure 1b–d). Their length (3–80 µm), width (0.3–3.0 µm), and apical branching increase from proximal to distal direction (Figure 1b; Figure S1, Supporting Information). The shortest un‐branched hairs are called spinulae (Figure S1a,b, Supporting Information) and appear also on the back and belly epidermis. The longer hairs with branched apices are called *setae* (Figure 1c,d; Figure S1c, Supporting Information). The tips of the setae are widened and flattened. They are called *spatulae* and have a wide of ≈250 nm (Figure S1e, Supporting Information).

Setae on the toe pad surface are organized in packs of four (Figure 1c,d). Scanning Electron Microscopy (SEM) images reveal that setae are formed by bundled fibrils with diameters 0.2–0.4 µm (Figure 1e; Figure S1f,g, Supporting Information). These fibrils emerge from the pad surface and assemble into a bundle along the seta stalk (Figure S1f, Supporting Information). *Setae* sprout at the apical side with a characteristic topical bristle structure (Figure 1d; Figure S1c,d, Supporting Information). The setae emerge out of an already fused and cornified keratinocyte layer (Figure 1e; Figure S1f, Supporting Information).

Setae contain α‐keratin and CBPs, as revealed by immunofluorescence staining (Figure S2, Supporting Information). α‐Keratin is found at the periphery of the setal cross‐section (Figure S2b, Supporting Information), whereas CBPs are observed on the inside (Figure S2d, Supporting Information). This core–shell distribution of CBPs and α‐keratins is in agreement with previous observations^[^ ^12^ ^]^ as well as with the general understanding of the role of these proteins as cornifying materials.

### 2.2 Membrane Shape Changes During Setae Development

**Figure 2:**
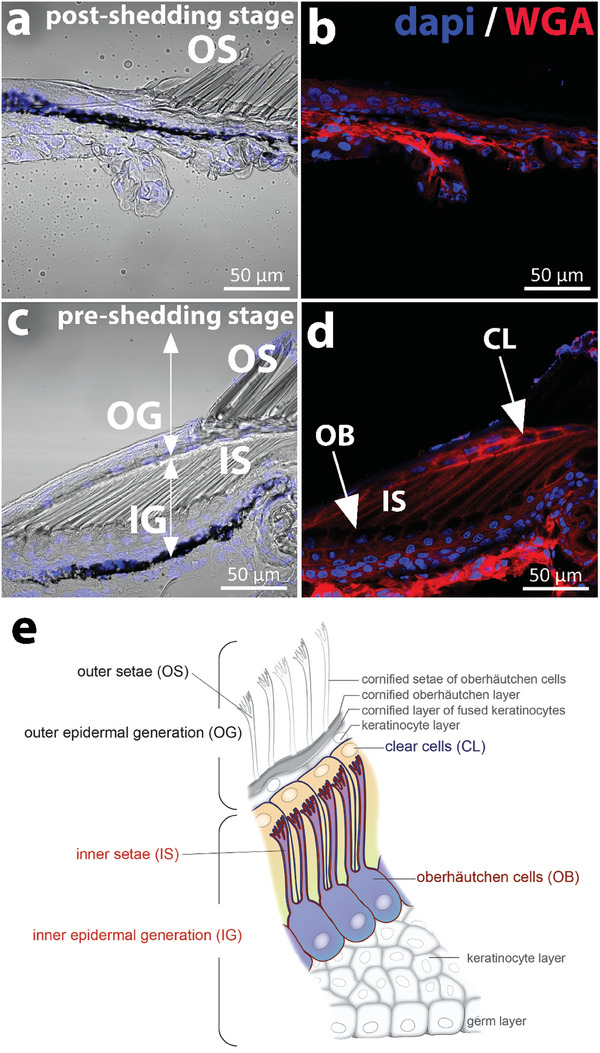
Longitudinal cryosectioned pad lamellae from geckos in two different shedding stages: a, b) post‐shedding c, d) and pre‐shedding imaged with Zeiss LSM880 (Plan‐Achromat 63x/1.4 Oil DIC M27). a) bright‐field image merged with nuclear staining (DAPI) of the post‐shedding tissue sample. The outer setae (OS) in the outer epidermis generation are indicated. b) Fluorescence image of (a) staining with rhodamine labeled wheat‐germ‐agglutinin (WGA) to visualize the cellular plasma membrane. c) Bright‐field image with nuclear staining (DAPI) of the pre‐shedding tissue sample. Two epidermal generations with the OS and the inner setae (IS) are visible. d) Fluorescence image of (c) staining with rhodamine labeled WGA to visualize the cellular plasma membrane. e) Scheme representing the cell layers in the toe pad epidermis in pre‐shedding strage. The shedding complex formed by the clear (CL) and oberhäutschen (OB) layers is highlighted. Illustration by IlluScientia, Dr. Agnieszka Kawska.

**Figure 3:**
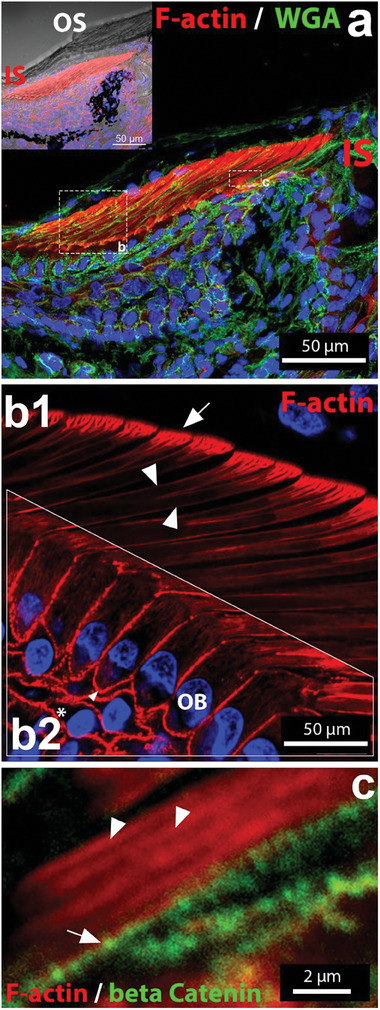
F actin structures in cross‐sections of toe pad lamella. a) General overview image of a longitudinal‐section of a whole toe pad lamella stained with Phalloidin‐Alexa 488 (red) and WGA‐rhodamine (green). The dashed boxes serve for orientation to the images (b) and (c). Inlay shows bright‐field image of OS (setae of outer epidermal generation) and IS (setae of inner epidermal generation). b) F‐actin structures within the IS from a longitudinal‐cryosected toe pad. F‐actin is present in the developing setae (b1) and as cortical and cytosolic actin in OB cells (b2). The keratinocytes in other epidermal layers (asterisk) show cortical actin. Staining: Phalloidin‐Alexa 546 (red). c) Longitudinal section of a IS stalk. Arrows show adherent junctions (β‐catenin, green) as dots. Arrowheads indicate F‐actin cables within IS.

New setae in lizards are formed during epidermal differentiation and morphogenesis of the new epidermal layer. To study the mechanism of setae development, we analyzed skin samples from two Bibron geckos at two different shedding stages: the first sample was just after shedding (post‐shedding) and the second sample was in a pre‐shedding stage. **Figure** **2**a–d shows longitudinal cryosections of a toe pad lamella. In both geckos, fully developed, cornified setae were observed at the outer surface (outer setae (OS) in Figure 2a,c,e). Skin samples at the post‐shedding stage showed the presence of a stratified epidermis below the cornified layers, with 2–3 layers of keratinocytes of similar shape and size (5–10 µm height and 10–15 µm width, Figure 2a,b, and **Figure** **3**b2). Skin samples at the pre‐shedding stage revealed the presence of two different epidermal generations (Figure 2c,d): the mature, outer generation (OG) with the cornified outer setae and other cornified layers, and a developing inner epidermal generation (IG) with developing, inner setae (IS). In the IG, one distinct keratinocyte layer (the top layer of IG) with an elongated shape could be distinguished after staining the cell membrane with WGA‐rhodamine (Figure 2d, also visible in Figure 3b). These hypertrophic cells form the Oberhäutchen (OB) layer, which interfaces with the clear (**CL)** cell layer (Figure 2d). OB cells have a cuboidal cell body shape and are significantly taller (≈23 × 8 µm) than the squamous keratinocytes in the underlying epidermis (≈14 × 8 µm, Figure 3b2). The nuclei of OB cells are located at the basolateral side and the basolateral membrane has a well‐defined rounded shape. Clear cells also have a cuboidal shape (≈25 µm height × 4.5 µm width) but their nuclei are located at the median/apical part of the cell (Figure S3a–c, Supporting Information). At the apical side of the OB cells long and parallel membrane protrusions with increasing length from proximal to distal (with ≈47 µm most distal; Figure 3a,b) were observed. These protrusions are developing setae according to reported literature.^[^ ^4^, ^13^ ^]^ A scheme of the different layers is represented in Figure 2e.

### 2.3 Actin and Tubulin in the OB Cells Drive IS Formation as Apical Protrusions and Template Setal Hierarchical Structure

The growth of apical structures in epithelial cells requires a core of bundled cytoskeletal filaments that actively deform the membrane.^[^ ^14^ ^]^ To reveal the cytoskeletal organization at the interface between the OB and clear cell layers, we stained different cytoskeletal components, i.e., actin, microtubules, and keratin intermediate filaments.

**Figure 4:**
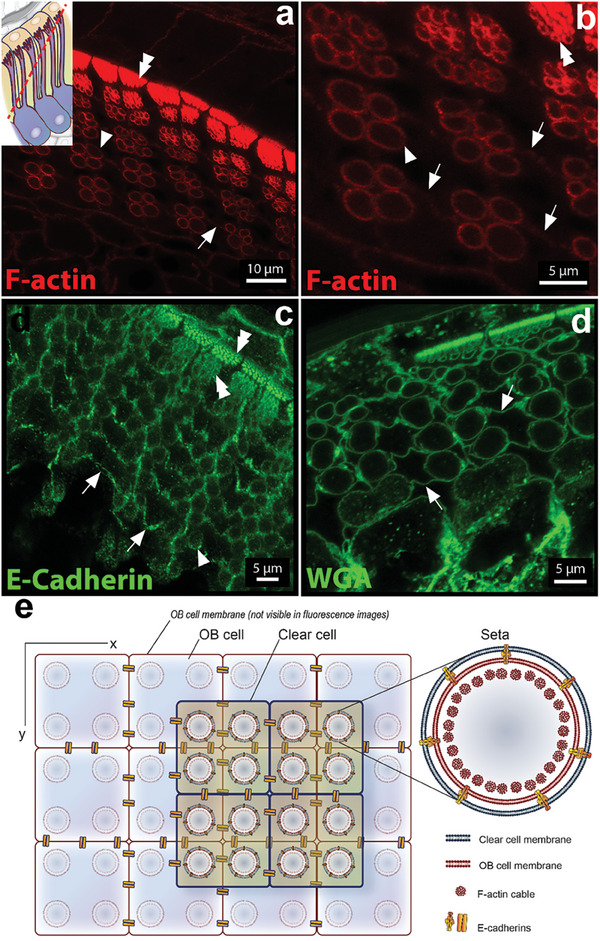
a–d) Immunofluorescence images of IS cross‐sections. a, b) Staining of F‐actin (red) reveals actin signal located at the setae periphery (arrowheads). Inlay in (a) shows the orientation of the cross‐section through the IS. c) Staining with E‐cadherin (green). Arrowheads indicate adherens junctions at the OB–CL cell membranes surrounding the setae. d) Staining of cell membrane (wheat germ agglutinin‐rhodamine). In (a–d) arrows indicate a line of fluorescence signal between the setae. In (a‐c) double arrowheads highlight the cell membrane around individual setae. e) Scheme representing the cross‐section imaged in (a–d) with the stained cellular components highlighted. Illustration by IlluScientia, Dr. Agnieszka Kawska.

Phalloidin staining revealed a very strong F‐actin signal in the protrusions of the OB cells compared to the rest of the keratinocytes in the epidermis (Figure 3a). F‐actin is organized in the form of long and branched structures with a hierarchical organization resembling mature setae (Figure 3b1,2). High‐resolution images of longitudinal sectioned samples revealed that the F‐actin structures are composed of fibrils (Figure 3c, arrowheads), which organize to form a tubular structure (**Figure** **4**a,b). F‐actin tubes extended along the entire length of the OB protrusions (Figure 3b1,2), i.e., from the stalk to the bristles of the developing setae. F‐actin tubes at the stalk had a diameter of ≈2.6 µm (Figure 3b2) and at the branches of ≈0.12 µm (estimated from SEM images of OS in Figure S1e, Supporting Information). The dimensions, uniformity, and filamentous and branched geometry of the tubular F‐actin structures appear analogous to the morphology of mature setae visible in the electron microscopy images in Figure 1 and Figure S1 (Supporting Information). Actin tubes were surrounded by cell membranes, as shown by *β*‐catenin staining in Figure 3c of cross‐sectioned IS. E‐cadherin staining confirms that the membranes of the OB and CL cells surround the entire IS, from the base (arrowheads in Figure 4c) to the tip (double arrowheads in Figure 4c). The observed F‐actin pattern indicates that F‐actin polymerization in part drives the formation of setae as apical protrusions from OB cells at the OB–CL interface (Figure 4a, inlay). This results in an interdigitated cell–cell interface formed by these two distinct cell layers (Figure 4e).

OB cells also showed a prominent actin cortex and a less pronounced longitudinal F‐actin network in the cytosol (Figure 3b2). Cell layers below the OB showed a strong cortical F‐actin and a weak cytosolic actin signal (asterisk in Figure 3b2).

**Figure 5:**
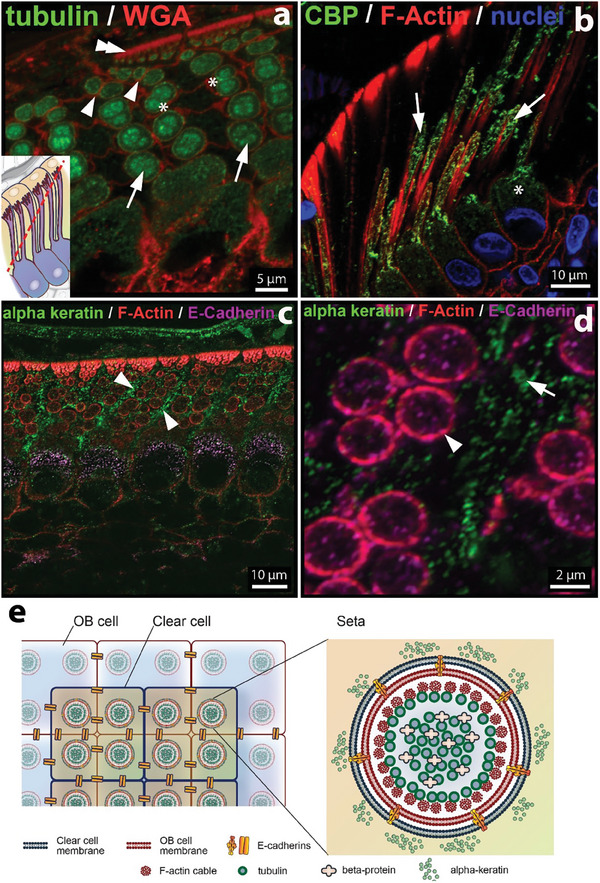
Cytoskeletal proteins in IS cross‐sections. a) Immunofluorescence images after Tubulin/WGA (green/red) labelling in cross‐sectioned IS near the base (arrows), at the onset of setal branching (asteriscs) and at the apical bristle of setae (arrow heads). Inlay in (a) gives orientation of the cross‐section through the IS. The membrane between the branches at the bristles is highlighted by the double arrows. Figure S5 shows the individual stainings. b) CBPs (green signal: Alexa 488) and F‐Actin (red signal: Phalloidin‐Alexa 546) in longitudinal sections. CBP is located inside setal core (arrow) and surrounded by peripheral F‐actin tubes. The asterisk highlights the OB cell body with CBPs in the cytosol. Nuclei are stained with DAPI c) α‐Keratin (green), F‐actin (red) and E‐cadherin (purple) staining. The α‐keratin (arrowhead) is located outside the setal lumen. d) Zoom‐in image of setal cross‐section. E‐cadherin signal colocalized with the setal actin tubes. Arrowhead indicates F‐actin tube. Arrow indicates α‐keratin outside of the setal lumen. e) Scheme representing the superposed OB and CL layers and the arrangement of the cytoskeletal structures inside the setae. Illustration by IlluScientia, Dr. Agnieszka Kawska.

We next examined a possible role of microtubules in setae formation, as they are a key building block of various cell protrusions, e.g., in apical cilia. Immunostaining with alpha‐tubulin antibody revealed an intense fluorescence signal within the individual F‐actin tubes (**Figure** **5**a, arrows). Similarly, immunolocalization of CBPs was found within the F‐actin tubes in developing setae, as dotty fluorescence spots localized along the entire setae length (Figure 5b, arrows). This pattern resembles that of *β*‐packets described in previous TEM studies. The dots were also found in the cytosol of the OB cell body. Note that CBPs are keratin‐associated proteins with a cementing role in keratin structures.^[^ ^8^, ^15^ ^]^ Taken together, these results suggest that actin and tubulin in OB cells serve as a template for the morphogenesis of setae, and their organization sets the basis for the deposition of CBPs as the main groups of proteins forming the setae. The assembly of these proteins determines the complex and hierarchical design of a gecko's toe pad surface. The dotty distribution of the CBPs signal suggests that these proteins accumulate in the setae for complete cornification at a later stage of the developing process. Experiments with samples closer to the shedding point are needed to confirm how keratin structures evolve during cornification.

### 2.4 The Contribution of the CL Cell's Cytoskeleton to Setae Formation

OB and CL are closely associated at the shedding interface. We therefore investigated the cytoskeletal organization at the cytosol of the CL. Immunofluorescent staining with alpha keratin did not detect alpha‐keratin inside or in the periphery of the actin tubes of the OB cells (Figure 5c,d, Figure S4, Supporting Information), although alpha‐keratin was present in the shell of the cornified outer setae (Figure S2b, Supporting Information). Instead, alpha keratin was found as a diffuse pattern in the space between the setae (arrowheads in Figure 5c; arrows in Figure 5d), suggesting that it was accumulated in a non‐polymerized form in the cytoplasm of CL cells. Longitudinal cryosections stained for α‐keratin also indicate a diffuse pattern of these intermediate filament proteins (Figure S4a, Supporting Information). Co‐staining the samples for E‐cadherin (arrowhead in Figure 5d and Figure S4k, Supporting Information) to mark intercellular adhesive contacts confirmed the cytosolic localization of alpha‐keratin in clear cells. The E‐cadherin signal (purple) and the F‐actin signal (red) colocalized at the setal periphery, indicating that the protruding setae are surrounded by the membranes of the OB and clear cells. α‐Keratin signal filled the space between the setae as part of the cytosol of CL cells (Figure 5c–e, single channels in Figure S4, Supporting Information).

In summary, the apical membrane of the OB and the basal membrane of the CL cell layer form an interdigitated interface, where cytoskeletal structures on the OB and the CL contribute to form new setae. F‐actin and microtubules drive membrane protrusions from the OB cell into the clear cell. Keratin accumulates in the cytosol of the CL and CBPs inside the actin tubes of the OB during setae development, and forms mechanically stable surface structures during cornification of the tissue, which occurs before shedding. This leads to a core‐shell structure of the cornified setae, where α‐keratin is located at the setae periphery and CBPs occupy the center.

### 2.5 Organization of Cell–Cell Junctions at the OB–CL Cell Interface

**Figure 6:**
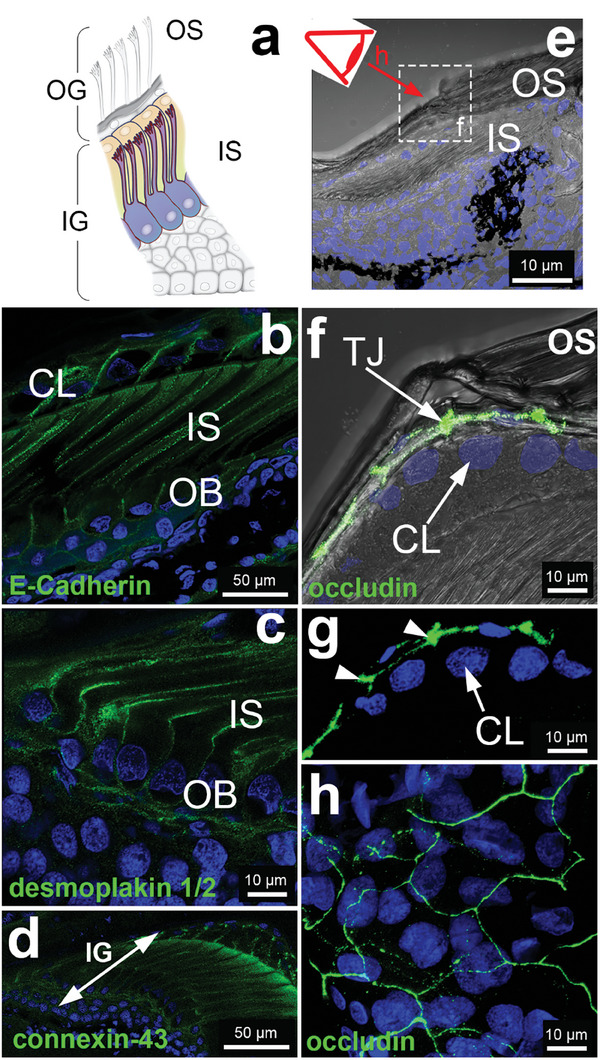
Immunofluorescence images of cell–cell contact proteins in longitudinal cryosections a) Scheme of a longitudinal sectioned toe pad epidermis with IG and OG for orientation; b) E‐cadherin (green) signal at the CL‐OB cell interface in longitudinal sections. c) Desmoplakin 1/2 (green) staining shows higher accumulation of desmosomes at the apical membrane of the OB cells, at the basis of the setae. Desmosomal fluorescence signal is also observed along the setae length. d) Connexin‐43 antibody (green) shows gap junctions along the setae length. e) Bright‐field overview for orientation for f, g, and h. dashed box indicated the area where (f) is taken. f) Merged bright‐field/fluorescence image of (g). g) Tight junctional marker occludin (green) in a longitudinal section. The arrowhead points to the last living keratinocyte layer of the OG that expresses the barrier forming tight junctions; h) Top view on toe pad after occludin staining showing a TJ bearing cell layer, orientation is highlighted with red eye in overview scheme (e). Nuclei are stained with DAPI. Illustration by IlluScientia, Dr. Agnieszka Kawska.

We expected that the transformations at the OB–CL cell interface during cornification and shedding affect the organization of cell–cell junctions at the cell membrane.^[^ ^16^ ^]^ Therefore, we studied the distribution of cell–cell junctions in the developing setae of the Bibron's gecko. Adherens junctions (AJs) were stained using E‐cadherin and β‐catenin antibodies. The corresponding signal was found along the entire setae length, from the base to the branched tips, and it colocalized with the cell membrane marker WGA (**Figure** **6**b; Figure S3 a–c, Supporting Information). This indicates that the interdigitated apical membrane of OB cells and the basal membrane of the clear cells are connected through AJs to maintain close contact during setae development. E‐cadherin and β‐catenin signals co‐localized with the F‐actin tubes, corroborating the linkage of the F‐actin cytoskeleton to E‐cadherin via β‐catenin at the membrane.

Desmosomes are anchoring points for the intermediate filament network and thus contribute to the stabilization of cytoskeletal structures within keratinocyte layers.^[^ ^17^ ^]^ Staining of Bibron´s gecko's epidermis with antibodies against desmoplakin isoforms 1 and 2, which link the keratin cytoskeleton to desmosomal cadherins, showed fluorescence signals along the entire OB cell membrane with the highest intensity located at the sub‐apical plane, from where setae protrude (Figure 6c). This distribution is in agreement with the observed localization of alpha‐keratin at the interspace between setae, in the cytosol of the clear cells.

Gap junctions (stained with connexin‐43 antibodies) were found along the entire membrane of the OB and clear cells and along the setae, indicating that intensive intercellular communication occurs between the two cell layers (Figure 6d). The highest fluorescence intensity was observed at the branched apices, probably due to their high branching. A few cell layers beneath the OB layer were also connexin‐43 positive, but layers above the clear layer were not. Interestingly, tissue samples from a different animal also in the pre‐shedding stage (Figure S3 e–g, Supporting Information) showed connexin‐43 positive cells only within a few keratinocyte layers underneath the OB cell layer. This result suggests that the concentration of gap junctions could vary during the self‐renewal cycle, with a loss of gap junctions in the new epidermal layer as the animal approaches the shedding point and the OB layer cornifies. For the verification of this hypothesis, additional experiments with animals at different shedding stages are needed.

Tight junctions (TJs, stained with occludin antibody) were neither found in the OB nor in the clear layer. Instead, TJs were found in the cell layer above the clear cell layer (Figure 6f,g). This layer is the last living keratinocyte layer of the outer generation (orientation in the epidermis in Figure 6f), and its cells have a flat phenotype that differs from the squamous morphology in the rest of the keratinocyte layers (Figure 6h).

### 2.6 Off‐Set Arrangement of OB and Clear Cell Layers: Four Setae Protrude from an OB Cell into Four Different Neighboring Cells in the Clear Layer

**Figure 7:**
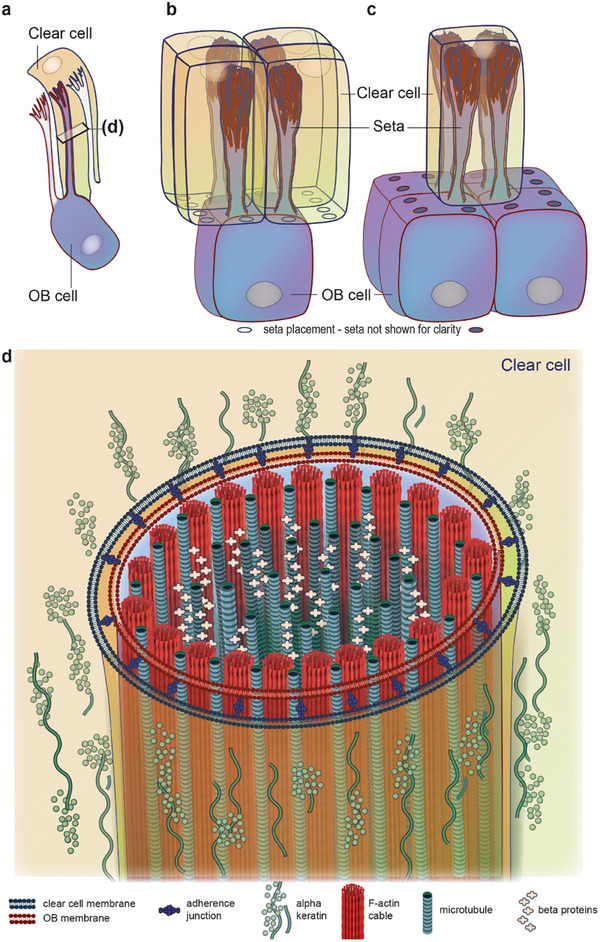
a) Representation of interdigitated OB (blue) and CL (yellow) cells and the IS. b) Representation of the spatial organization of CL cells with respect to a single underlying OB cell. The four setae of the OB cell protrude into four different neighboring CL cells. c) Representation of a CL cell containing the setae protruding from four different underlying OB cells d) 3D scheme of a cross sectioned seta stalk with all analyzed cellular components. Illustration by IlluScientia, Dr. Agnieszka Kawska.

According to SEM images (Figure 1c,d) of the OS on the toe pad surface, setae are arranged in groups of four in a square‐like format. The fluorescence images of cross sections of IS at the stalk plane also show groups of four actin tubes (Figure 4a,b, arrowheads) and corroborate that this arrangement is already present in the IS. In the cross‐sections, the cortical actin signal (Figure 4a,b, arrows), the E‐cadherin signal (Figure 4c), and the membrane signal (Figure 4d) of neighboring clear cells are found in between the four setae of the pack. This indicates that the packs of four setae, which protrude from a single OB cell, penetrate four different neighboring CL cells. These observations suggest that each setae of the pack is situated in a different CL cell (**Figure** **7**b.c). This image is compatible with an offset arrangement of OB and clear cells within their cell layers, which has been described in previous works with anolids.^[^ ^18^ ^]^ In summary, the fluorescence images indicate that a clear cell harbors four setae from four different, adjacent OB cells underneath (Figure 7b,c). Note that in this configuration, the four setae of the OB cell would be separated by multicellular junctions. These are spatial landmarks with distinct functions in the control of geometry and topology of epithelial tissues during development,^[^ ^19^ ^]^ and as hot spots of epithelial tension.^[^ ^19b^ ^]^ We hypothesize that a multicellular junction separating neighboring setae at the shedding plane could have a relevant role in the stabilization of the setae and/or during the shedding of the outer epidermal layer.

## 3 Discussion

The present study describes the morphology of developing setae as apical protrusions in keratinocytes. In such structures, actin and microtubules are expected to reorganize the cell membrane into its required functional shape, while intermediate filaments take a stabilizing role during elongation.^[^ ^1a^ ^]^ In a similar way, the geometry of the gecko's setae seems to be templated by F‐actin and microtubules inside intercellular membrane protrusions at early developmental stages, onto which keratins and CBPs assemble. In differentiated OB cells in developing setae from a Bibrons gecko at the pre‐shedding stage, we found keratin intermediate filaments and CBPs arranged in cytoplasmic particles but not in extended filaments or bundles. In contrast, in mature setae analyzed by SEM, these proteins are found as long filamentous structures. Our data suggest that keratin and CBP filaments in setae form during the cornification of the tissue, and likely follow the template provided by F‐actin and tubulin. We acknowledge in support of this hypothesis that a templating role of F‐actin in the assembly of cytokeratin networks has also been observed in cell‐free extracts.^[^ ^20^ ^]^

Setae apical protrusions form at the interface between two cell layers. While the interdigitated morphology of the OB–CL cell interface is unique, finger‐like protrusions and invaginations at cell–cell interfaces have been observed in other epithelia. For example, in simple epithelial monolayers interdigitated membrane structures at the lateral plane have been suggested to contribute to cell–cell adhesion and tissue integrity,^[^ ^21^ ^]^ Cryptic lamellipodia are thought to sustain cell–cell contact surfaces in collective migrating epithelial cells.^[^ ^22^ ^]^ Intercellular membrane protrusions are also relevant for cell competition and cell extrusion in epithelial tissue homeostasis and disease.^[^ ^21^, ^23^ ^]^

The distribution of cell–cell junctions at the OB–CL cell interface shows similarities with other epithelial cell layers. Puncta‐like patterns of adherens junctions stained by E‐cadherin in the gecko's epidermis have also been identified as protruding microspikes in epithelial cell layers.^[^ ^24^ ^]^ These are considered to represent a mechanism that reinforces intercellular adhesion by generating an interlocking membrane topography that increases the cell–cell contact surface area to promote cadherin–cadherin adhesive interactions. Actin protrusive activity helps to push the lateral membranes of neighboring cells together and to keep cadherins in contact.^[^ ^24b^ ^]^ The accumulation of desmoplakin at the subapical plane is in agreement with its localization in other epithelial protrusions. Desmoplakin has been proven relevant to maintaining the shape and length of microvilli in intestinal epithelium,^[^ ^25^ ^]^ with a presumed contribution to the stabilization of the terminal web onto which the actin rootlets of microvilli are inserted. Plakin cytolinkers and keratins also control the length of epithelial microridge protrusions.^[^ ^1a^ ^]^ In skin epithelium, loss of desmoplakin enhanced protrusiveness and led to an increase in filopodia length and number, both in monolayers and single cells.^[^ ^26^ ^]^ During the cornification of the mammalian epidermis, desmoplakin together with keratins becomes covalently crosslinked into the cornified envelope and stabilizes the structure.^[^ ^17^ ^]^ The distribution of desmoplakin in the gecko's skin could indicate a similar role.

From an engineering point of view, the high surface area that develops at the OB–CL cell interface during setae formation appears disadvantageous for shedding, since the high aspect ratio of the protrusions leads to strong shear forces during peeling off that could damage the geometry of the setae.^[^ ^27^ ^]^ In this context, the multicellular junctions in the CL cell that separates the four protruding setae from the OB cell could serve as a tension hotspot and vulnerable structures,^[^ ^28^ ^]^ which facilitate disintegration and peeling off the dead skin layer without damaging the new setae. This hypothesis needs confirmation in tissue samples right at the shedding point.

Setae are not the only functional high‐aspect ratio skin appendages that develop from membrane extensions. Adhesive structures in frogs also involve elongated and directionally organized subcellular structures.^[^ ^29^ ^]^ In feathers, barbule cells elongate by the accumulation of parallel bundles of CBPs and microtubuli, though the mechanism has not been investigated.^[^ ^30^ ^]^ Drosophila's mechanosensitive bristles also evolve as membrane protrusions, 250–400 µm in length and 5 µm width, by directional polymerization of F‐actin and microtubules in specialized cells.^[^ ^31^ ^]^ Understanding how such functional surface structures self‐form in natural tissues can open new avenues to bioengineer self‐morphing structures of unprecedented complexity in the future, as well as to more sustainable approaches to generate high‐performance functional materials.^[^ ^32^ ^]^ Apical protrusions in epithelial monolayers like the distinct adhesive setae in gecko skin, fit into this vision. With the ability to shape cells in vitro or in alternative synthetic multicellular surrogates using reconstituted bionic cells,^[^ ^33^ ^]^ emerging technologies for novel materials that can self‐form, reconfigure, or adapt in response to stimuli are imaginable. Such approaches could replace micro‐ and nanostructuring processes with synthetic photoresist materials, elastomeric replicas, or carbon nanotubes, as examples of gecko mimics designed to control the grip and movement of robots on earth and in aerospace.^[^ ^2^, ^34^ ^]^

A limitation of the current study is that tissue from only three Bibron's thick‐toed geckos was used. Future work with tissue in different stages, across more individuals and species will be required to see how broadly the results of this study apply across geckos.

## 4 Experimental Section

### Gecko Source

Three Bibron´s thick‐toed geckos (*Chondrodactylus bibronii*) were purchased from an authorized pet shop. They were euthanized in accordance with the German legislation on the protection of animals. For euthanasia, the animals were kept in their preferred optimal temperature zone (22–28 °C) and pentobarbital sodium was injected intracoelomial. Subsequent decapitation was conducted. The experiments with *C. bibronii* in this study are compliant with the Animal Welfare Laboratory Animal Ordinance as well as with the Nagoya‐Protocol ((EU) Nr. 511/2014). One gecko showed only the outer epidermal generation and was categorized as to be in the post‐shedding stage. The two other geckos showed an outer and an inner epidermal generation with well‐developed IS. These samples were categorized to be in a pre‐shedding stage. The tissues from the post‐shedding stage gecko were used in Figure 3a,b.

### Tissue Preparation and Sectioning

To prepare for sectioning at the cryostat, parts of the toe samples were snap frozen, embedded in cryoglue, and kept at −80°C for 30 min until cryoglue completely solidified. Other parts were excised and prefixed with 4% paraformaldehyde (PFA) in phosphate‐buffered saline (PBS) for 24 h at 4 °C. These samples underwent a sucrose gradient (10% sucrose in PBS for 24 h, 25% sucrose in PBS for 24 h at 4°C) and were then embedded in cryoglue and kept at −80°C. Embedded toe pads were taken to cryostat at −20°C for 5 min to adjust temperature. The thickness of the section was 10 µm. Sections were mounted on histobond slides and if not prefixed they were fixed immediately with 4% PFA in PBS at room temperature (RT) for 10 min. After fixation sections were washed 3× with tris‐buffered saline (TBS).

### Scanning Electron Microscopy (SEM):

SEM (FEI Quanta 400 FEG; FEI Deutschland GmbH, Frankfurt, Germany) was used without conductive coating in low vacuum mode at p_H2O_ = 100 Pa) to analyze the surface of an air‐dried foot of a bibron's thick‐toed gecko *Chondrodactylus bibronii* in its natural state after freeze fracture of a digit in liquid nitrogen and snap frozen cryosections of a digit.

### Immunofluorescence Staining and Imaging

Sections on histobond‐slides were permeabilized with 1% Triton X 100 in TBS for 10 min at RT and subsequently washed 3× with TBS. To remove PFA remnants, samples were incubated with ammonium chloride (NH_4_Cl_2_: 1 mm in water) for 5 min at RT. Samples were washed 3× with TBS and incubated with 1% TBSA (bovine serum albumin in TBS for 30 min for blocking. All primary antibodies were diluted 1:50–1:100 in TBSA and incubated for 1 h at RT. After incubation, samples were washed 3× with TBSx 0.1% (TBS with 0.1% Triton X 100), and secondary antibodies were applied 1:1000 in TBSA 1% for a further 1 h at RT in the dark. Then, samples were washed again with 3× TBSx 0.1% and further 3× with TBS. Stained slides were finally mounted with mounting media (ProLong Gold). As required, phalloidin Alexa‐488/546 or WGA‐rhodamine was added 1:100 to the secondary antibody dilution. Imaging was conducted with the confocal laser scanning microscope Zeiss LSM 880 with Airyscan. Following primary antibodies were used: β‐catenin: BD 610154; E‐cadherin: antibodiesonline ABIN1077673; α‐tubulin: antibodiesonline ABIN152113; CBPs general antibody (pre‐corebox): Davids Biotechnology, peptide sequence: TSAASLGILSGASPSCINQI; desmoplaquin 1/2: Progen 61 003; connexin‐43: BD Bioscience 610061; occludin: Santa Cruz, sc‐271842. Following secondary antibodies were used: 1. (IgG (H+L) Highly Cross‐Adsorbed Donkey anti‐Mouse, Alexa Fluor 488, Invitrogen; Fisher Scientific Cat.: 10544773; IgG (H+L) Highly Cross‐Adsorbed Donkey anti‐Mouse, Alexa Fluor 546, Invitrogen; Fisher Scientific Cat.: 10698093; Donkey anti‐Rabbit IgG (H+L) Highly Cross‐Adsorbed Secondary Antibody, Alexa Fluor 594, Invitrogen; Fisher Scientific Cat.: 10798994. Following fluorescence dyes were used: Invitrogen Alexa Fluor 488 Phalloidin, Fisher Scientific Cat.: 10125092 or Invitrogen Alexa Fluor 546 Phalloidin, Fisher Scientific Cat.: A22283; Wheat Germ Agglutinin, Tetramethylrhodamine Conjugate, Fisher Scientific Cat.: W849; nuclei are stained with DAPI 4′,6‐Diamidine‐2′‐phenylindole dihydrochloride, Sigma–Aldrich Cat.: 10236276001.

### Fluorescence Confocal Imaging and Image Processing

The fluorescence images were acquired with a Zeiss LSM 880 confocal microscope with an Airyscan detector and Plan‐Apochromat 63×/1.4 Oil DIC M27 objective. Images were opened in Zeiss Zen light software (Zen 3.4 blue edition). The brightness and contrast of each image was checked and adjusted to optimum for each channel if required. The image was exported as TIFF using the export function in Zeiss Zen light software. Some images were cropped using the cropping tool in Adobe Photoshop CS6, for better fitting in the respective Figure compilation (Figures 3c and 6g,d). Figure compilations were conducted using Adobe Illustrator and Photoshop (Adobe Suite CS6).

## Conflict of Interest

The authors declare no conflict of interest.

## Author Contributions

J.Y.K. performed the conceptualization, methodology, validation, formal analysis, and investigation stages, additionally, wrote, reviewed, and edited the original manuscript, also, contributed to visualization efforts and played a key role in project administration. M.W.L. performed supervision and investigation, wrote, reviewed and edited the final draft. M.K. performed the investigation. L.A. performed the supervision, reviewed and edited the final draft. T.M. performed the supervision, wrote, reviewed and edited the final draft. C.M.N. performed the supervision, wrote, reviewed and edited the final draft. A.d.C. performed the conceptualization, supervision, project administration, managed resources, acquired funds, wrote the original manuscript, and wrote, reviewed and edited the final draft.

## Supporting information

Supporting Information

## Data Availability

The data that support the findings of this study are available from the corresponding author upon reasonable request.
