# Supplementary material for: Actin‐templated Structures: Nature's Way to Hierarchical Surface Patterns (Gecko's Setae as Case Study)
Source: Adv Sci (Weinh). 2023 Dec 25;11(10):2303816. doi: 10.1002/advs.202303816 (PMC10933612; doi:10.1002/advs.202303816)
Supplement: Supplementary file 1 — Supporting Information [file ADVS-11-2303816-s001.pdf]

ADVANCED SCIENCE
Open Access

Supporting Information

for *Adv. Sci.*, DOI 10.1002/advs.202303816

Actin-templated Structures: Nature's Way to Hierarchical Surface Patterns (Gecko's Setae as Case Study)

Jennifer Y. Kasper, Matthias W. Laschke, Marcus Koch, Lorenzo Alibardi, Thomas Magin, Carien M. Niessen and Aránzazu del Campo**

Actin-templated structures: nature's way to hierarchical surface patterns (gecko's setae as case study)

Jennifer Y. Kasper [1,*], Matthias W. Laschke [2], Marcus Koch [1], Lorenzo Alibardi [3], Thomas Magin [4], Carien M. Niessen [5], Aránzazu del Campo [1,6,*]

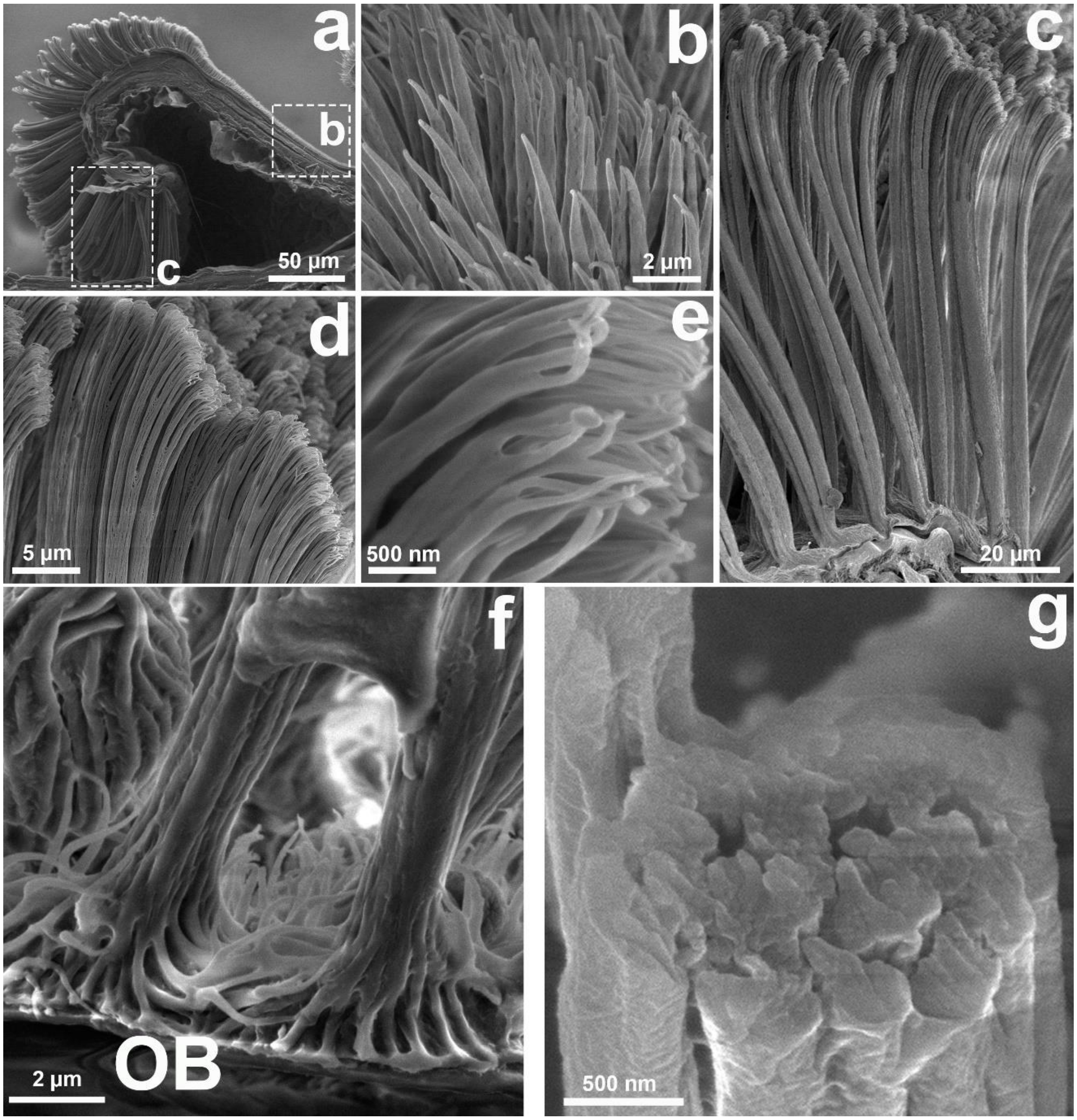

Fig. S1. Scanning electron micrographs of the setae of Bibron´s thick-toed gecko (*Chondrodactylus bibronii*) **(a)** Overview picture of longitudinal sectioned toe pad and view on the section plane. Dashed boxes are magnified in (b) and (c). **(b)** Spinulae (side view), which are observed most proximal on toe pad. **(c)** Setae observed most distal. **(d)** Higher magnification of the highly branched setal apex. **(e)** Higher magnification of the apex with spatulae. **(f)** Higher magnification of the base of two mature setae from a longitudinal section including the cornified Oberhäutchen cell. **(g)** Cross-cryosectioned mature setae that displays the inner morphology.

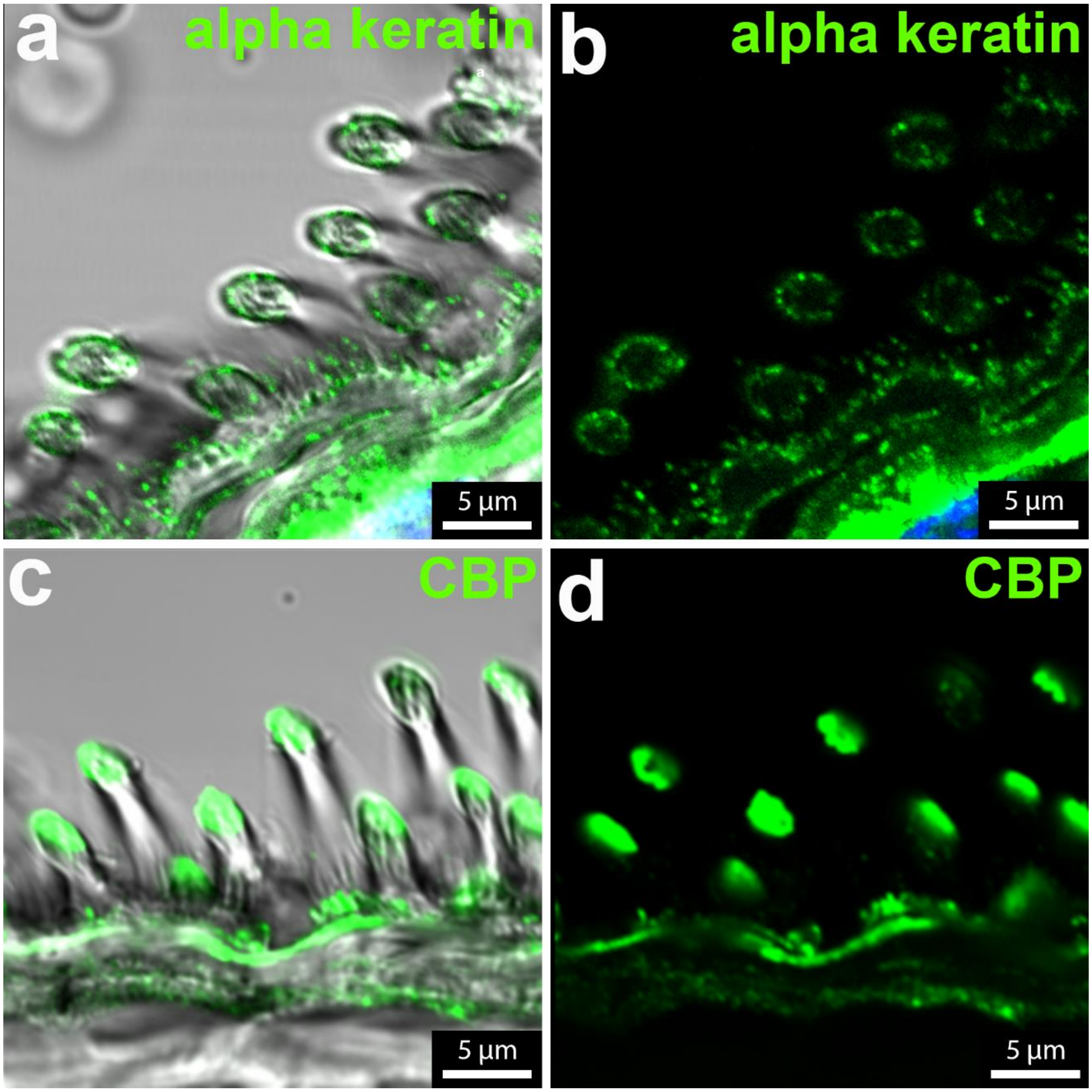

Fig. S2. Bright-field merged with immunofluorescence (IF) confocal images of cryostat sections showing cross-sectioned setae of the outer epidermal generation stained for alpha-keratin **(a,b)** and CBPs **(c,d)**.

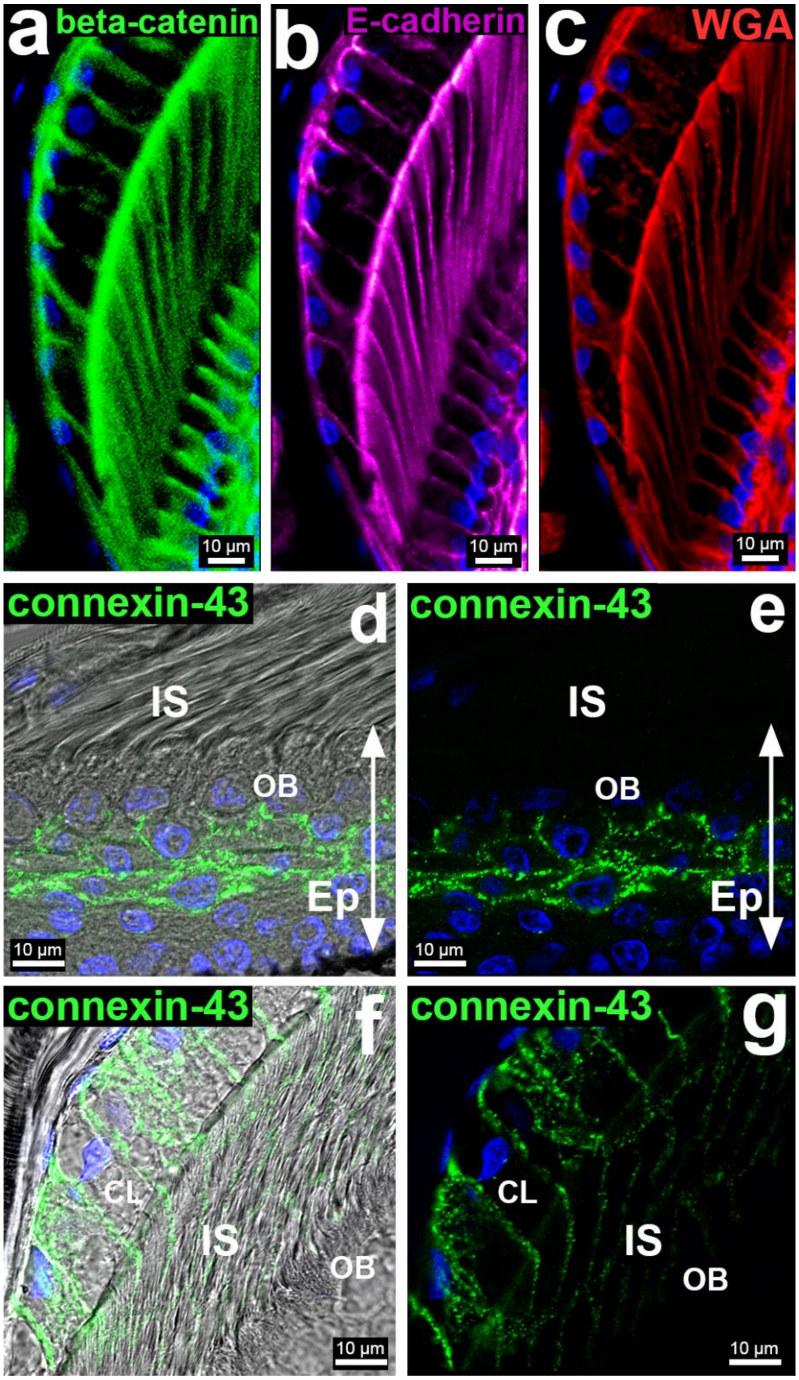

Fig. S3: **(a-c)** IF confocal images of longitudinally cryosectioned IS. **(a)** Beta-Catenin (green); **(b)** E-Cadherin (purple); **(c)** cell membrane (red, WGA-rhodamine). **(d)** Bright-field overview image of

(e) connexin-43 staining (green). **(f)** Bright-field overview image of **(g).** IF of connexin 43 in another gecko in different shedding stage: ep: Epidermis. Nuceli are stained with dapi

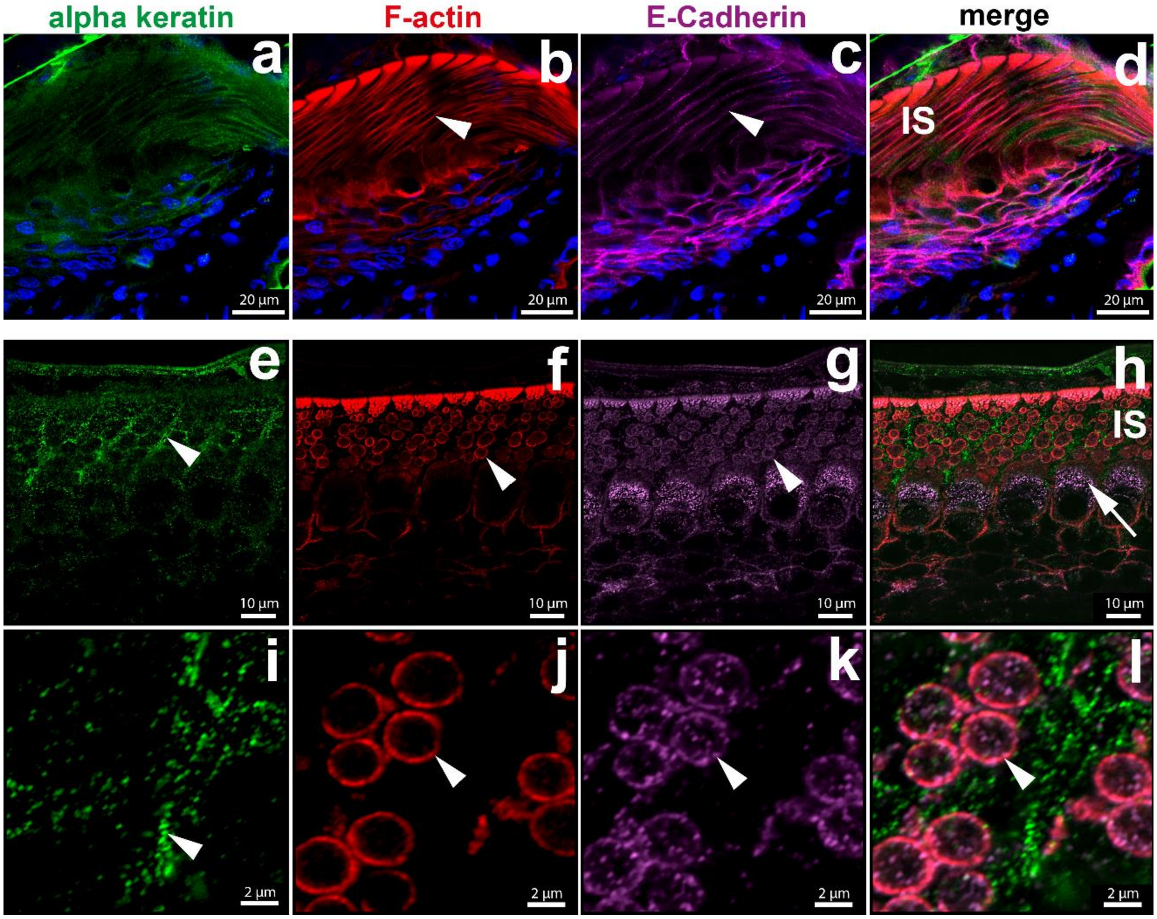

Fig. S4: IF images of alpha-keratin (green: pan Cytokeratin), F-Actin (red: Phalloidin-Alexa 546) and E-Cadherin (purple). **(a-d)** Longitudinal cryostat sections of IS. Arrowhead in b indicates actin tubes in close proximity to the E-Cadherin-labelled cell membrane (arrowhead in c). **(e-h)** Cross sections of IS. **(i-l)** higher magnification of (e-h). Arrowhead in (e) and (i) indicates alpha keratin located outside the setal lumen. E-Cadherin highlights cell borders (arrowheads in g, k and l). F-actin bundles are located in the setal perifery close to the E-Cadherin labelled cell membrane (arrowheads in j,k). Furthermore, a dotty E-Cadherin signal appears in the OB cells (arrow in h) as well setae lumen. E-Cadherin positive cell borders are located around the periphery of the actin tubes in IS(arrowhead in j-l), Nuclei are stained with Dapi (blue)

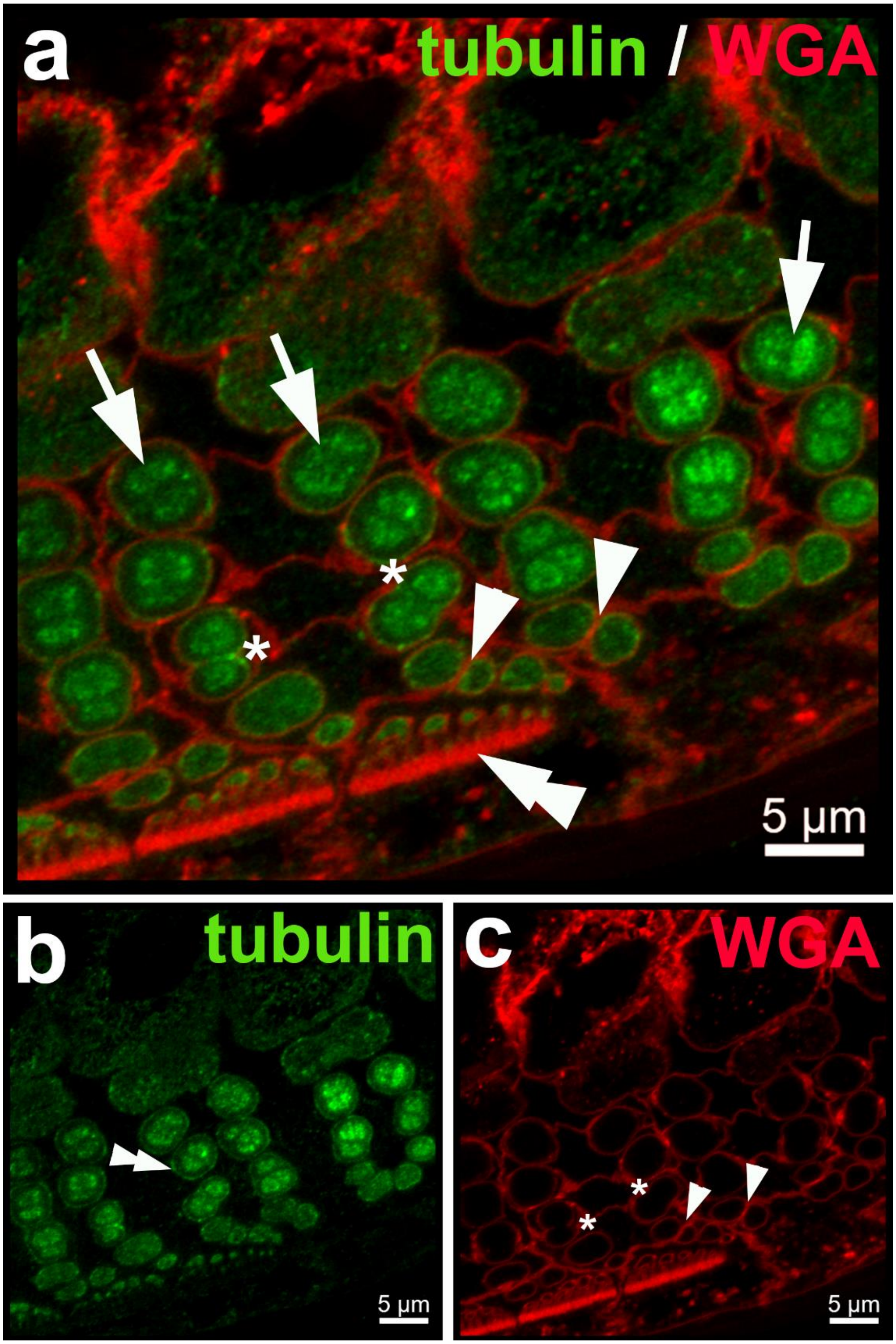

Fig. S5: Tubulin/WGA (green/red) immunofluorescence IF staining of cross sected IS near its base corressponding to Figure 3a in the manuscript. **(a)** Merged image. **b)** Green (tubulin) channel. **(c)** Red WGA-rhodamine staining. Nuceli are stained with dapi

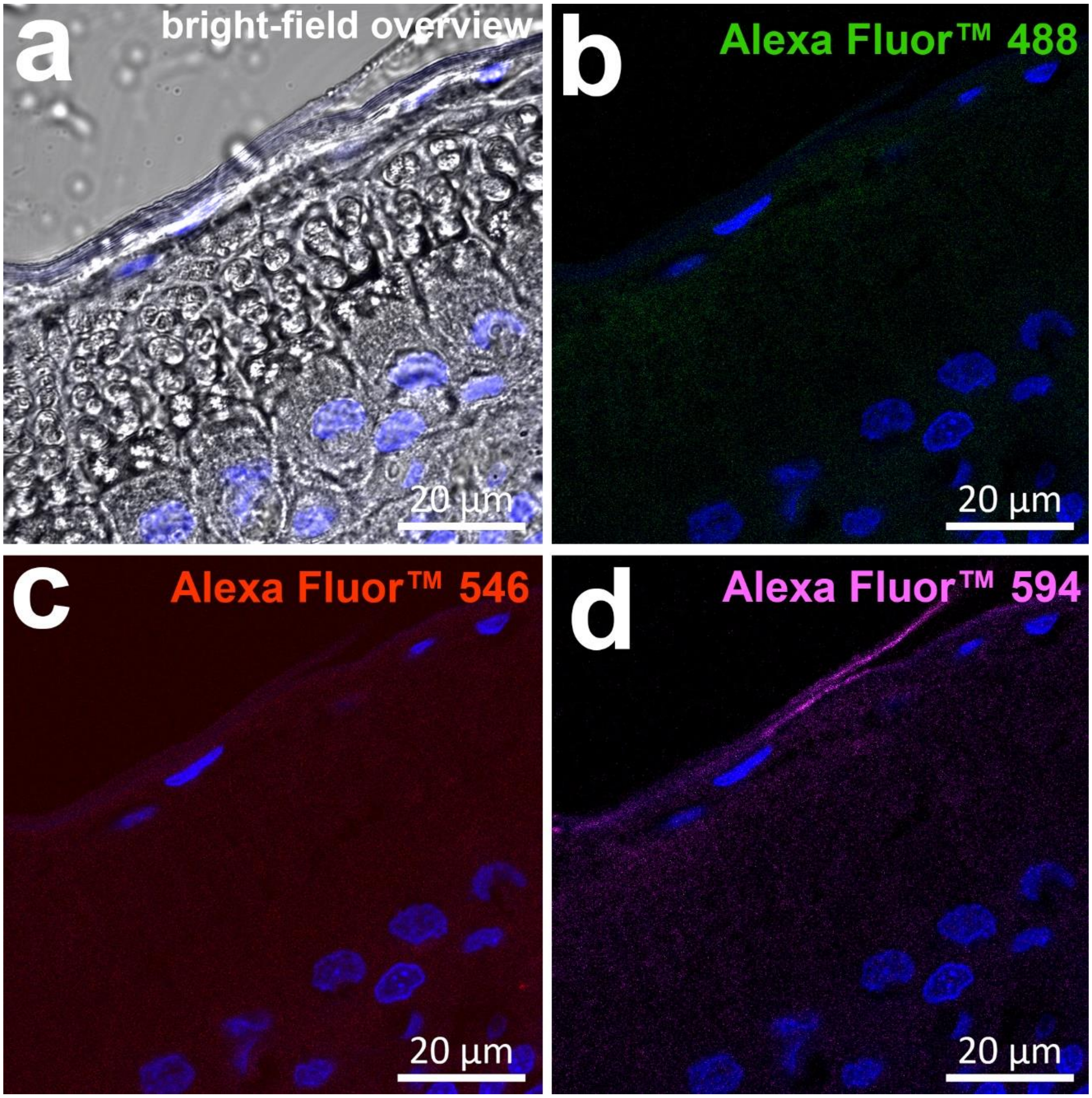

Fig. S6: IF of secondary antibody controls without primary antibody **(a)** bright-field overview merged with dapi **(b)** green channel 488 (IgG (H+L) Highly Cross-Adsorbed Donkey anti-Mouse, Alexa Fluor® 488, Invitrogen™; Fisher Scientific Cat.: 10544773); **(c)** red channel 543 (IgG (H+L) Highly Cross-Adsorbed Donkey anti-Mouse, Alexa Fluor® 546, Invitrogen™; Fisher Scientific Cat.: 10698093); **(d)** purple channel 594 (Donkey anti-Rabbit IgG (H+L) Highly Cross-Adsorbed Secondary Antibody, Alexa Fluor™ 594, Invitrogen™; Fisher Scientific Cat.: 10798994); nuclei are stained with dapi. The secondary antibodies used in this study do not show significant cross-reactivity in gecko epidermal tissue samples. Only Alexa Fluor™ 594 showed a small unspecific signal in the outer cornified keratin layer. Therefore, this antibody was only used to answer specific questions where e.g. a fourth channel was needed and did not interfere with E-cadherin immunofluorescence stainings e.g. **Figure 3 and S3**
